# Reliable evaluation of left ventricular function during veno-arterial extracorporeal membrane oxygenetion

**DOI:** 10.1186/2197-425X-3-S1-A541

**Published:** 2015-10-01

**Authors:** P Morimont, P Massion, G Hans, J Guiot, T Desaive, A Pironet, B Lambermont

**Affiliations:** University Hospital of Liège, Intensive Care Unit, Liège, Belgium; Department of Anesthesiology, University Hospital of Liège, Liège, Belgium; University of Liège, Faculty of Sciences, Liège, Belgium

## Introduction

Precise assessment of left ventricular (LV) contractility during veno-arterial extracorporeal membrane oxygenation (VA-ECMO) is crucial. However, changes in loading conditions may mask changes in LV function when assessed with load-dependent indices like LV ejection fraction (LVEF). Indeed, these indices do not reflect intrinsic LV contractility but are the result of interaction between preload, contractile function and afterload [1].

## Objectives

The aim of this study was to show that precise assessment of LV function with a load-independent index is feasible during VA-ECMO.

## Methods

We used end-systolic elastance (Emax), which is the load-independent reference parameter corresponding to the maximum elastance of the left ventricle, to determine LV intrinsic contractility [2]. We combined invasive arterial pressure and LV volume derived from echocardiography, at several pre- and after- load levels resulting from changes in VA-ECMO flow, to determine Emax. This analysis was performed on a 54-year-old man supported with femoro-femoral VA-ECMO for cardiogenic shock. Afterload was calculated by arterial elastance (Ea) as the ratio of end-systolic pressure to stroke volume (SV). We compared LVEF and Emax from day 1 to day 3.

## Results

Emax increased from 0.51 to 1.09 mmHg/mL, LVEF decreased from 17 to 14%, SV decreased from 27 to 20 mL and Ea increased from 2.63 to 4.45 mmHg/mL.

## Conclusions

While LVEF and SV decreased because of increased afterload, LV function assessed with Emax revealed an improvement in LV contractility. Emax is more reliable than LVEF to assess LV function when the loading conditions are changing. This load-independent parameter may be determined simply by using changes in VA-ECMO flow.

## Grant Acknowledgment

Leon Fredericq Foundation of the University of Liège, Belgium
